# Investigating attributes for selecting nurse sows in swine herds of Minnesota, USA, using Best-Worst Scaling analysis

**DOI:** 10.1016/j.vas.2026.100656

**Published:** 2026-04-12

**Authors:** Joab Malanda Osotsi, Renata Knop, Samira Chatila, Pedro Urriola, Peter Balogh, Gabriella-Novotni-Danko

**Affiliations:** a Department of Animal Science, Institute of Animal Science, Biotechnology and Natural Conservation, Faculty of Agricultural and Food Sciences and Environmental Management, University of Debrecen, Boszormenyi Street 138, 4032 Debrecen, Hungary; b Doctoral School of Animal Science, University of Debrecen, Boszormenyi Street 138, 4032 Debrecen, Hungary; c College of Food, Agricultural and Natural Resource Sciences, University of Minnesota Twin Cities, St Paul MN 55108, United States; d Faculty of Economics and Business, University of Debrecen, Boszormenyi Street 138, 4032 Debrecen, Hungary

**Keywords:** Large litters, Nurse sow selection, Managers preferences, Discrete choice experiment, Conditional Logit Model

## Abstract

Nurse sow selection is aimed at ensuring ideal sows are chosen to manage surplus piglets in hyper prolific herds. This study investigated attributes farm managers preferred when choosing nurse sows. We evaluated seven attributes: Body Condition Score (BCS), Sows' Current Litter Size (SCLS), Sows' Current Litter Health (SCLH), Sow Behaviour (SB), Sow Teat Number (STN), Lactation Stage (LS), and Parity (P). Choice decisions of 51 managers were stratified into five subgroups: gender, age, pig-farming experience, education level, and herd size managed. We used Best Worst Scaling analysis to evaluate relative importance of attributes, while Conditional Logit Model assessed their utility importance, setting P as the baseline. Results showed that SCLH was the most important while P the least important. All six attributes were significantly preferred over the baseline. Female managers showed significant preferences for all attributes than male managers. Younger managers (18–40 years old) placed significant preference on sow physical qualities compared to older managers (> 40 years old). Managers with ≤10 years’ experience demonstrated wider significant preference profiles than those of >10 years. Managers with college degree preferred half of the attributes while those with professional degrees (BSc/MSc) gave significant relevance to almost all attributes. Managers managing herds of up to 5000 sows viewed all attributes significant while those in charge of extra-large herds (>5000 sows) gave no preference to SB and LS. This research provides new empirical evidence and nuanced analysis of nurse sow selection, placing SCLH as a priority proxy for selecting a nurse sow.

## Highlights

• Hyper prolific herds require effective nurse-sow selection for surplus piglets.
• We identified important attributes driving farm managers’ choices of nurse sows.
• Sow body condition and pig health influenced managers’ preferences.
• Young female managers preferred a wider range of traits.
• Education and experience influenced the strength of attribute preferences.

**Implications**

We investigated pig farm managers preferences in selecting nurse sows for managing large litters. Managers preferred health and body condition traits to behavioural traits. Demographic stratification appeared to shape decision-making process. In pig husbandry, considering managers selection preferences can enhance the adoption of sow management strategies. The findings would therefore improve knowledge on nurse sow selection and successful piglet fostering.

Alt-text: Unlabelled box dummy alt text

## 1 Introduction

Hyper prolificacy has gained genetic momentum in modern pig farming, leading to large litters that are challenging for the sow's udder, especially when live born exceeds available functional teats. For instance, hyper prolific sows can farrow up to 18–20 live born piglets, yet functional teats are limited to an average of 14 (Vande Pol et al., 2021; Earnhardt-San et al., 2023). Data from the National Agricultural Statistics Service of the US Department of Agriculture shows that the average number of pigs per litter increased by 1.10% annually between 1993 and 2020, resulting in a 36.2% increase in litter size (United States Department of Agriculture 2025). Regardless of whether the litter is born alive or stillborn, large litters are defined as having more piglets than the sow can nurse with her functional teats.

Several methods have been investigated to raise the surplus piglets in an effort to manage large litter size. Strategies such as (i) artificial rearing where piglets are raised in special feeding systems (Pustal et al., 2015) (ii) double nursing where a sow nurses two litters in rotation from farrowing till weaning (Houben et al., 2017), and (iii) the use of nurse sows where a sow that has just weaned a litter is given surplus piglets to nurse extending her lactation period (Baxter et al., 2013; Bruun et al., 2016). In intensive large-scale pig operations, management of these surplus piglets is made possible by the use of nurse sows or artificial rearing. Nurse sows help in maximizing pre-weaning survival, particularly for low-birth-weight piglets (Craig et al., 2019), and improve average daily gain together with weight at weaning (Rezac et al., 2023). Sows used as nurse sows take longer in lactation helping their uterus to involute in preparation for subsequent pregnancy (Rezac et al., 2023).

Effective nurse sow management begins with careful selection from the lactating sow group. Because random selection can compromise sow and piglet performance, intentionally choosing nurse sows based on specific criteria is essential to the success of the system. Although farm practices vary across regions and even among individual farms, several common considerations have been identified. For example, first and second parity sows are favorably chosen because of their small teats, which make them more suitable for small piglets (Weber et al., 2009). In a ranking of qualities for nurse sow selection in Denmark, surveyed pig farmers reported considering sow body condition score, parity, the number of functional teats, current litter condition, previous nursing ability, and the number of weaned piglets (Bruun et al., 2016, 2023; Sorensen et al., 2016). An expert review assessing economic aspects of using nurse sows in Sweden found that factors for selecting nurse sows included parity, lactation stage and sow robustness (Alvasen et al., 2017). Developing a deeper understanding of nurse sow selection may enable farm managers to develop appropriate tools and dissemination methods to meet their individual farm scenario.

Best-Worst Scaling (BWS) also called maximum difference scaling (maxdiff) is a type of discrete choice experiment where participants are shown a number of option sets, each set including a subset of all items that need to be assessed (Auger et al., 2007). It falls under conjoint analysis methods, which identifies preferences and trade-offs that influence people's decisions (Lancsar et al., 2008; Cohen 2009). It examines the position of a set of attributes along a subjective dimension that is assumed to exist. Examples of these subjective dimensions include the degree of relevance and the degree of interest. In the degree of relevance the attribute is evaluated on how applicable it is while in the degree of interest the attribute is evaluated on how appealing it is to the respondent. This is done by measuring respondent preferences out of at least three aspects rather than rating each aspect separately (Mühlbacher et al., 2016). The items in a choice set are chosen by respondents in a way that maximizes their differences on an underlying scale of significance (Erdem & Rigby 2013), enabling them to make trade-offs. This method enables respondents to choose the best (most important) and the worst (least important) attributes using a series of repeated choice sets (Flynn et al., 2007). BWS makes it possible to gather data from a smaller sample (Jourdain et al., 2022) and does not reduce the respondents' cognitive ability (Jourdain et al., 2022).

The BWS method has been widely used primarily in marketing research with few applications in livestock production as follows. In the U.S., BWS was used to identify individual preferences for sustainable beef practices and policies among US beef producers (Osman et al., 2024). In Europe, BWS was utilized to assess the preferences of European dairy farmers on breeding for resilient and efficient dairy cattle (Burns et al., 2022), and the economic worth of animal welfare choices among Swedish dairy farmers (Hansson et al., 2016). In the UK, BWS has been used to assess UK sheep producers’ decisions on agricultural greenhouse mitigation measures (Jones et al., 2013). Here, farmers shared their preferences on mitigation decisions that effectively reduce greenhouse gas emissions in sheep industry. Applying Best-Worst Scaling in pig production would therefore allow for a better understanding of how farm managers prioritize on qualities for choosing nurse sows.

Against this background, the objective of this study was to investigate how swine farm managers in Minnesota, the United States of America (U.S.A.), preferred different attributes for selecting nurse sows in trade-off situations using the Best–Worst Scaling (BWS) analysis. This study contributes to the field of pig husbandry in two ways: (i) the BWS method may be utilized to support decision-making in pig production systems. (ii) the results may contribute to the advancement of knowledge about nursing systems by promoting the use of discrete choice experiment for nurse sow selection in trade-off situations.

## 2 Materials and methods

### 2.1 Description of the study area

The study surveyed pig farm managers in Minnesota, USA. Minnesota is one of the leading pig-producing states in the US with approximately 9.2 to 9.4 million pigs as of late 2025, ranked 2nd after Iowa state (United States Department of Agriculture 2025). Pork is Minnesota number one livestock export and the state marketed about 16.73 million pigs in 2023 generating $12.53 billion in total economic output from about 3000 pig farms (Minnesota Pork Board 2025). Farm sizes are classified into small (< 2 000 sows, n = 1685), medium (2 000–4 999 sows, n = 824), and large (≥ 5 000 sows, n = 419) (United States Department of Agriculture-National Agricultural Statistics Service, Census of Agriculture, 2022). Swine farming in this state is largely characterized by large operations / Concentrated Animal Feeding Operations (CFAOs) comprising about 65 % of Minnesota’s pig inventory (United States Department of Agriculture-National Agricultural Statistics Service, Census of Agriculture, 2022). These commercial operations can either be (i) farrow-wean systems where sows produce piglets which are sold to finishing operations, (ii) finishing systems that raise weaned pigs till market weight, and (iii) farrow-finish systems, which cover all phases of breeding till marketing. The latest available data indicate that there were 157 farrow-wean farms, 611 farrow-finish farms, and 2073 finishing farms in Minnesota (United States Department of Agriculture-National Agricultural Statistics Service, Census of Agriculture, 2022). Based on these data, the number of farm managers overseeing farrow-wean systems in Minnesota can be estimated to be approximately 157, assuming that each farm is managed by at least one site manager. For this investigation, written consent was obtained from a swine company in Midwest USA that oversees various farrow- wean farms. Their farm managers were requested to participate in the survey. The choice of farm managers as the appropriate study respondents was informed by the following reasons (i) they are in charge of making decisions at the farm level; (ii) in most cases, they have typically risen through the ranks and gained substantial experience working on one or more farms; and (iii) they are able to train lower cadre staff members in a variety of management aspects.

### 2.2 Sampling design

We first conducted an online pilot study with eleven (11) farm managers. Eighteen (18) attributes for selecting nurse sows were identified from the review of Osotsi et al. (2024). Managers had to select which ones they considered in choosing nurse sows. These factors covered aspects relating to sows, piglets, and farm management. According to the pilot study, Body Condition Score (BCS) was ranked top in the sow category, while Sows Current Litter Size (SCLS) was the highest in the piglet-related category. Biosecurity concerns and breed targets were lowly rated as management related. We used the findings in the pilot study to select the attributes to be considered in the next phase of BWS discrete choice experiment.

We chose seven attributes for evaluation based on the pre-trial study's highest rank scores as recommended by Webb et al. (2021) for discrete choice experiments. However, most of those attributes were found to have been mentioned as relevant factors in selecting nurse sows in studies conducted primarily in Denmark (Bruun et al., 2016, 2023;Sorensen et al., 2016). The attributes were as follows; Body Condition Score (BCS), Parity (P), Sow Behaviour (SB), Sow’s Teat Number (STN) and Lactation Stage (LS) as sow related and Sow’s Current Litter Size (SCLS), Sow’s Current Litter Health (SCLH) as piglet related. The description of these attributes is provided in Table 1.

**Table 1:** Description of attributes featured in the BWS discrete choice experiment.

Attribute	Description
Body Condition Score (BCS)	Is an attribute that evaluates a pig’s overall fat and muscle reserves. Is crucial for assessing a sow's general health, reproductive capacity, and nutritional status.
Sow’s Current Litter Size (SCLS)	Indicates the number of piglets a sow is nursing at the time of selection. This number may not be its litter size at farrowing.
Sow’s Current Litter Health (SCLH)	Depicts the piglets' general well-being and health at the time of sow selection. Implies vitality, vigor and absence of disease.
Parity (P)	The number of times a sow has farrowed. Serves as an indicator of the sows' level of output and reproductive history.
Sow Behavior (SB)	It includes a sow's overall temperament, maternal instincts, and receptivity. Most depicted during farrowing and lactation.
Sow’s Teat Number (STN)	The number of functional teats available for suckling.
Lactation stage (LS)	Refers to a sow's stage of milk production. Usually divided into three phases: early, mid, and late lactation (about 0–7 d, 8–14 d and 15–21 or 28 d, respectively).

A Balanced Incomplete Block Design (BIBD) (Salinas Ruízet al. 2024) was utilized to construct the BWS choice sets (Louviere et al., 2015). A BIBD ensures that each attribute appears the same number of times across tasks (equal occurrence) and is paired with every other attribute an equal number of times (equal co-occurrence). This lessens the possibility that respondents would inadvertently deduce information about the objects from the design elements. The number of objects in each set and the number of subsets is intended to effectively and repeatedly capture data regarding each respondent's relative preferences. This design organizes items into option sets where each item appears alongside other items a certain number of times (Louviere et al., 2015). The BIBD for v characteristics is represented by the following notation: b, r, k, λ, where r is the repetition per level, k is the number of items in each choice set, and λ is the number of times each pair of items appears together in the same choice set. λ is the pair frequency, and b is the number of choice sets (Cohen 2009). Our study's design was 7,3,3,1 for seven attributes (selection criteria) and seven choice sets. Each choice set had three attributes, and each attribute appeared once alongside the others. We selected a minimal set size of three attributes because we believed that a set size of four and above would make the choice task cognitive demanding for participants (Cohen 2009). Furthermore, we maintained our BIBD at the minimal required level because participant boredom sets in when they are shown >10–12 choice sets (Cohen 2009). An example of the BIBD experimental design used is shown in Table 2.

**Table 2:** An example of BIBD choice set.

How important are the following factors for you when selecting a nurse sow.		
From the following three factors, please indicate which one is the Most Important and which one is the Least Important		
**Attribute**	**Most important**	**Least important**
Body Condition Score	X	
Parity		
Sow Teat Number		X

Respondents were asked to select the most and least important aspect for each situation according to what they preferred when choosing a nurse sow. Their decision was marked with an X.

### 2.3 Data collection

From June 2024 to September 2024 we employed an online quantitative survey where farm managers filled the survey. The survey was designed with two sections: (i) Demographic section: This section collected information about age, education level, gender, and years of experience working on a pig farm. (ii) The second and main section employed the BWS. The survey was written in English, and a Spanish translation was included for each statement (Supplementary file 1). At the time of investigation, the production company operated 71 farrow-wean herds, each managed by a site manager. Managers were not pre-selected to participate in the survey, and the authors had no prior relationships with the respondents that could have influenced their participation. Participants received a Google form link of the survey through their respective work email address. The email had a brief introduction informing managers the purpose of the study and requesting them to participate. The email was sent to 71 managers and 51 responded within the study period. Participation was entirely voluntary and anonymous. Respondents did not receive compensation for taking part in the survey. We adhered with accepted ethical standards as well as data protection of participants. Responses were automatically saved in the Google Forms database and retrieved as a CSV file for analysis.

### 2.4 Statistical analysis

#### 2.4.1 Best-Worst Scaling (BWS)

Data from the BWS were transformed into individual case-by-case for analysis. We counted the total number of times each attribute of interest was selected as best and worst across all sets aggregated for all individuals. Each scenario's best and worst options were tallied and converted into a best-worst score. An attribute overall score was determined by the net frequency of B − W scores.

The following formula was used to calculate BW value.

(1) BestWorstvalue=BestScore−WorstScore

Standard Best Worst values were then calculated using the second equation.

(2) $$Standard\;value=\frac{\left(BWS-Min\;BWS\right)}{\left(Max\;BWS-Min\;BWS\right)}$$

The square root of the BWS value was calculated using the third equation.

(3) $$Sqrt.BW\;value=\sqrt{\frac{Most\;important}{Least\;Important}}$$

Each attribute's relative importance was evaluated on a 0–100% scale using the following formula:

(4) $$Relative\;importance=\frac{\left(BWS\;value-Min\;\right)}{\left(Max\;-Min\;\right)}x\;100$$

A descriptive aggregate ranking was established based on relative importance.

Bar charts were created from the BWS scores associated with each attribute. Preference levels ranged from −3 (least preferred) to +3 (most preferred) and were displayed on the x-axis, while the frequency of each attribute was displayed on the y-axis. A score value of zero indicated that an attribute was either never selected as "best" or "worst," or that it was chosen as "best" just as frequently as it was chosen as "worst."

#### 2.4.2 Conditional Logit Model (CLM)

The Conditional Logit Model was used to calculate the chance an attribute will be chosen as best or worst based on every potential combination providing its utility importance (Osman et al., 2024). The model used the lowest ranked (least important) attribute as the baseline (reference category) to estimate the utilities of the remaining attributes in order to prevent perfect multicollinearity (Osman et al., 2024). In our phase 1 (BWS analysis), parity was the least important categorical variable and therefore used as a baseline with a utility score of zero. This made sure that every other attribute utility score was positive, reflecting how they were important and by how much in relation to parity. Therefore, in the first step of the CLM, we estimated utility coefficients of the remaining six attributes and compared to parity. In the second step, we explored heterogeneity in attribute preferences maintaining parity as the baseline. Because of the limitation in the number of respondents we received, we were unable to conduct latent class analysis. We decided to conduct subgroup analyses instead. Five sub groups each with two categories of demographic factors served as the basis for stratification.

(i) Gender (female vs male)
(ii) Age (18–40 years vs. over 40 years),
(iii) Years of experience in pig farming (up to 10 years vs. >10 years),
(iv) Educational attainment (college degree vs. professional degree (BSc or MSc)), and
(v) Sow herd size managed (up to 5000 sows vs. >5000 sows).

The experimental design of the BWS part (BIBD) was planned using the *crossdes* package of the R program (R Core Team 2020; Sailer, 2023). The data analysis of the BWS part was performed using the *support.BWS* and *Apollo* packages of the R program (R Core Team 2020; Aizaki & Fogarty, 2023; Aizaki 2023; Hess & Palma 2019, 2024). Statistical significance was assessed at a threshold of p < 0.05.

## 3 Results

### 3.1 Demographic characteristics of farm managers

Table 3 summarizes the demographic profile of farm managers based on stratified sub groups

**Table 3:** Demographic characteristics.

Sub group	Category	Number (%)	Total (%)
Gender	Female	18 (35.29)	51 (100)
	Male	33 (64.71)	
Age	18–40 years old	24 (47.06)	51 (100)
	Above 40 years old	27 (52.94)	
Experience	Up to 10 years	26 (50.98)	51 (100)
	>10 years	25 (49.02)	
Level of education	Up to college degree	15 (29.41)	51 (100)
	Professional training (BSc/MSc)	36 (70.59)	
Herd size managed	Up to 5000 sows	19 (37.25)	51 (100)
	>5000 sows	32 (62.75)	

### 3.2 Overall attribute ranking based on BWS relative importance

Table 4 shows the aggregate results of the BWS analysis. Each attribute's most and least important choices, BWS scores, standard values, rank order, and relative percentages are shown.

**Table 4:** Results of the BWS counting approach.

Attribute	SCLH	BCS	SCLS	STN	LS	SB	P
Most Important value	29.41	23.81	15.69	12.04	7.00	7.00	5.04
Least Important Value	4.48	5.60	15.13	14.85	15.97	17.97	26.61
BWS value	89	65	2	−10	−32	−37	−77
Standard value	0.58	0.42	0.01	−0.07	−0.21	−0.24	−0.50
Square root of Most/Least Important	2.56	2.06	1.02	0.90	0.66	0.64	0.44
Relative %	100	80.47	39.75	35.16	25.85	24.79	16.99
Final Rank	1	2	3	4	5	6	7

**Fig. 1:**
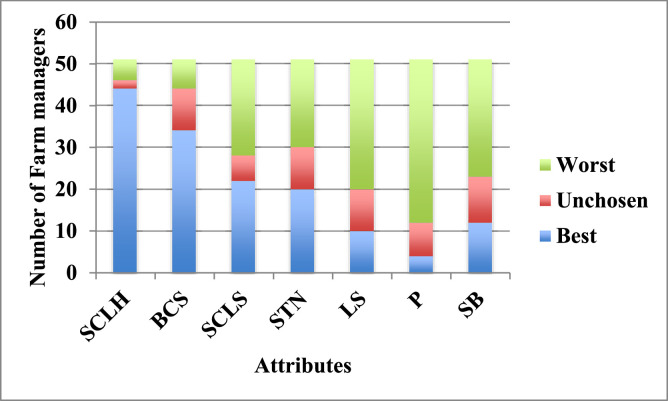
Frequency of attribute selected as Best, Unchosen or Worst.

Fig. 1 shows the number of times an attribute was selected as ‘Best’, ‘Unchosen’ or ‘Worst’ by farm managers across BWS choice tasks.

Higher counts in the “Best” category represent attributes deemed most important, whereas higher counts in the “Worst” category represent attributes regarded as less important.

### 3.3 Conditional Logit Model

**Table 5:** Overall results of conditional Logit Model estimates.

Attributes examined and estimated model data	CL estimates	
	Coefficients	Robust Standard Error
Sow’s Current Litter Health (SCLH)	1.744 ***	0.239
Body Condition Score (BCS)	1.455 ***	0.207
Sow’s Current Litter Size (SCLS)	0.811 ***	0.185
Sow’s Teat Number (STN)	0.697 ***	0.183
Lactation Stage (LS)	0.485 **	0.183
Sow Behavior (SB)	0.431 *	0.188
Parity (P)	Reference category	
Number of observations	357	
Pseudo R^2^	0.1492	
Log-likelihood (0)	−639.7	
Log-likelihood (converged)	−544.2	
AIC	1100	
BIC	1124	

Note: *** =p < 0.001, **=p < 0.01 and *=p < 0.05, AIC Akaike Information Criteria, BIC Bayesian Information Criteria.

### 3.4 Sub group CLM preference analysis

**Table 6:** Results of CL subgroup preference analysis.

			Attributes					
Sub group	Category	CL estimates	SCLH	BCS	SCLS	STN	LS	SB
Gender	Female (n = 18)	Coefficients	2.133***	1.767***	1.070***	0.929**	0.818**	0.637*
		RSE	0.421	0.399	0.294	0.305	0.308	0.313
	Male (n = 33)	Coefficients	1.562***	1.310***	0.691**	0.590*	0.324 NS	0.336 NS
		RSE	0.289	0.240	0.235	0.230	0.226	0.238
Age	18–40 years old (n = 24)	Coefficients	1.748***	1.516***	0.753**	0.816**	0.571 NS	0.374 NS
		RSE	0.372	0.317	0.261	0.269	0.295	0.280
	Above 40 years old (n = 27)	Coefficients	1.748***	1.408***	0.866**	0.593*	0.409 NS	0.481 NS
		RSE	0.314	0.279	0.267	0.255	0.233	0.259
Experience	Up to 10 years (n = 26)	Coefficients	1.905***	1.731***	0.698**	1.068***	0.701**	0.494 NS
		RSE	0.291	0.329	0.237	0.287	0.246	0.277
	>10 years (n = 25)	Coefficients	1.634***	1.228***	0.955**	0.342 NS	0.283 NS	0.382 NS
		RSE	0.394	0.277	0.304	0.237	0.276	0.268
Level of education	Up to college degree (n = 15)	Coefficients	1.237*	1.094**	1.095*	0.210 NS	0.108 NS	0.473 NS
		RSE	0.514	0.363	0.489	0.334	0.407	0.366
	Professional training (BSc/MSc) (n = 36)	Coefficients	2.048***	1.668***	0.717***	0.932***	0.666***	0.423 NS
		RSE	0.245	0.263	0.198	0.223	0.194	0.225
Herd size managed	Up to 5000 sows (n = 19)	Coefficients	2.068***	1.744***	1.425***	0.714*	0.718*	0.989**
		RSE	0.447	0.331	0.346	0.350	0.349	0.307
	>5000 sow (n = 32)	Coefficients	1.617***	1.344***	0.503*	0.720**	0.383 NS	0.136 NS
		RSE	0.293	0.273	0.212	0.225	0.219	0.289

Note: *** =p < 0.001, **=p < 0.01 and *=p < 0.05, NS Not Significant, RSE Robust Standard Error.

## 4 Discussion

In BWS analysis, we noted a distinct hierarchy of qualities, with SCLH and BCS appearing as the most important factors (Table 4). Decision-making in farm husbandry is critical because it has a direct relationship with farm performance (Taramuel-Taramuel et al., 2023). Managers prioritized quick, observable features that had a direct impact on piglet survival and growth, as evidenced by the strong preference for real-time elements of sow and piglet health. The importance of SCLH is consistent with pig management research that highlights the significance of animal health for both welfare and productivity (Kittawornrat & Zimmerman 2011). SCLH has a direct impact on piglet survival and performance, and the attention paid to it supports its practical relevance in fostering (Muns, 2016). Likewise, BWS research on consumer preference for pig meat consumption found that consumers consistently emphasized health and welfare-related aspects as the primary determinants of buying pork (Jaeger et al., 2008). Our findings provide an alignment of both producer and consumer priorities pertaining to pig health. Managers' preference for BCS is a prognosis for sow longevity and lactation performance. Both SCLH and BCS are factors providing useful information about the productivity of sows both at present and in the future. Our findings are in part consistent with other studies in dairy systems that used preference-based approaches for trait selection. Burns et al. (2022) study in dairy found that farmers gave more selection weight to traits associated with functional efficiency and resilience as compared to purely structural traits when designing their breeding index. Moderate importance was given to mid-ranked attributes such as STN and SCLS. Managers may view them as secondary to urgent health-related measures, even though both are crucial for successful nursing and piglet survival. This may stem from the fact that individual farm procedures may already have standardized what is optimal for such traits. The lowest ranks were given to attributes such as LS, SB, and particularly P. LS affects nurse sow- piglet acceptance and successful nursing. For instance, sows that were 21days in lactation when recruited as nurse sows showed slower nurse piglet acceptance and piglets experienced much udder competition (Schmitt et al., 2019). However, in our study, managers appeared to focus more on observable features than on physiological features. SB is paramount because instances of piglet directed aggression or rejection have been reported when nurse sows are given nurse piglets (Kobek-Kjeldager et al., 2020; Bortolozzo et al., 2023). The low preference for P can be the result of decision shifting its focus from past reproductive performance to present health and physical condition.

In CLM analysis, managers prioritized immediate maternal and piglet health indicators, as evidenced by their high utility estimates (Table 5). Earlier studies have also established the significance of litter condition and sow vitality as benchmarks for successful fostering (Alexopoulos et al., 2018). Litter health is an influencing factor for pre-weaning survival and sow fitness for cross fostering (Calderon et al., 2018). Strong, viable, healthy litters are a direct proxy for good mothering ability, which is necessary for the successful nursing of adopted piglets. The high preference for BCS is supported by Skorjanc et al. (2008) and Ajay et al. (2023) due to its correlation with health condition and lactation performance. Sows with good body reserves could have a better chance of sustaining prolonged lactation, a common feature in nurse systems. Furthermore, sows with adequate BCS have greater litter performance and reduced chances of postpartum problems (Ajay et al., 2023). Considerable preference for attributes such as SCLS and STN indicates that physical ability to nurse indicated by the number of functioning teats and litter capacity, is a practical issue in selection. Udder quality and teat functionality are essential for effective nursing and piglet survival. Nurse piglets must not exceed sows’ functional teats. Having a higher nurse litter and lower number of functional teats would inevitably result in nursed piglets missing a teat. Utility estimates for LS and SB were lower although these attributes were not completely overlooked. Their low preference may be due to focusing on physical rather than physiological traits, and the challenge of reliably evaluating sow behavior in commercial settings (Marchant-Forde 2015). Parity is an assessment of reproductive history and was consistently ranked as the least important, indicating that it would not supersede functional and observable qualities when selecting nurse sows. This finding is somewhat contrary to Lavery et al. (2019) who mentioned that parity was a major predictor of maternal performance. However, our study findings are supported by Baxter et al. (2024), who indicated that individual sow characteristics at the moment of fostering are better predictors of performance than parity.

In gender subgroup analysis, female managers preferred all six attributes to parity (Table 6). SCLH was the most valued attribute indicating that litter vitality and health conditions play a key role in nursing decision-making. Kristjanson et al. (2014) found that women frequently prioritize animal care and offspring wellbeing when making livestock decisions. In addition to health, emphasis on maternal strength was evidenced by the preference for BCS. Preferences for SCLS and STN were moderate but significant. Female managers appeared to show a balanced grasp of nursing needs and emphasize both the physical ability for nursing, as depicted by the number of teats, and the demand for litter size. Despite being ranked lower, LS and SB showed statistical significance. Female managers valued behavioral and physiological factors, although to them these had smaller influence than direct indicators of piglet health and sow condition. This finding is in part agreement with Manfre et al. (2013), who highlighted that in gendered agricultural roles; females are more sensitive to animal welfare and behavior in their management of livestock.

Male managers preferred SCLH and BCS, indicating that physical condition and indicators of health are universally acknowledged as being crucial by both sexes. STN was slightly significant, and SCLS continued to have a moderate influence. Male managers may prioritize broader maternal indicators than nursing capacity. In some studies where males are dominant in livestock decision-making, it has been shown that they would prefer to conform to a more quantitative selection centered on overall health and productive potential (Kristjanson et al., 2014; Ragasa et al., 2013). Males did not prioritize SB and LS, as seen in females. It has been observed that men in livestock production tend to prioritize performance-based attributes over nuanced indicators (Manfre et al., 2013). The above gender-based differences could provide a basis for customizing decision-support tools to aid decision-making frameworks.

In relation to age analysis, younger farm managers (18–40 years old) preferred SCLH and BCS (Table 6) depicting a reliance on quantifiable and performance-based selection cues. This demographic also prioritized both the maternal ability to nurse and piglet demand, as seen by the preferences for STN and SCLS. The demand (litter size) and supply (teat availability) equilibrium preference may be borrowed from productivity mindset echoed in European Union Commission (2025) report on Strategy for Generational Renewal in Agriculture Report, where the policy expects young farmers to be more market-oriented aligning with production and demand. Among this group, LS and SB were not preferred. This might be a result of less focus on physiological subtleties and behavioral characteristics.

Managers aged above 40 years also prioritized SCLH and BCS, demonstrating the universal prioritization of health and condition. SCLS was preferred among this group; however, STN only had a slight influence, which might be due to the focus on overall health rather than the exact sow teat capacity. Neither LS nor SB was statistically significant. This is similar to the younger group and somewhat surprising because, according to Morgan-Davies et al. (2012), older managers are believed to have more observational experiences and stockmanship skills. Therefore, in this study, they could be at an advantage in picking subtle observations than younger managers. Nevertheless, older managers may rely on habitual decision-making rather than subtle cues. The capacity to regularly monitor behavior could also be affected by context, such as greater herd sizes, time limits, and labor shortages. Age may impact the attention paid to specific secondary attributes, but the fundamental preferences for physical appearance and health are the same across all age groups.

On experience, managers with less than or equal to ten years of working experience favored SCLH and BCS, a clear emphasis on observable performance traits (Table 6). This group had a high preference for STN, underscoring the awareness of sows' capacity to accommodate piglets. Some production systems may advocate retooling and technical training for their workforce. Such training may emphasize structural and reproductive soundness. The moderate but considerable preference for SCLS and LS indicates an increasing awareness of the features of nursing integration. In this group, SB was not statistically significant. In time constrained farming operations, managers may not accurately assess behavioral indicators. This may result in oversight of behavioral cues, despite their potential to impact sow-piglet social interactions.

Managers with >10 years of experience affirmed their importance to SCLH and BCS. SCLS was substantially preferred, suggesting that managers still consider litter size a key factor, particularly when balancing suckling and supply capacity. STN, LS, and SB, on the other hand, were not preferred in this group, depicting a slightly different pattern from managers with <10 years of working experience. Experienced farmers tend to create intuitive or instinctive strategies based on cumulative observations over time that could help them in decision-making (Nuthall & Old 2018). Experience may encourage a streamlined focus, luring managers to prioritize productivity at the expense of other traits. In large-scale swine farms, observational limitations may be the cause of the lack of importance of SB.

Education wise, managers with at least a college degree ranked BCS as the most important attribute, followed by SCLH and SCLS (Table 6). This group seemingly favored physical indications of sow as crucial for lactation performance. The remaining half of the attributes, failed to gain preference. This might be as a result of training that emphasizes quantifiable performance traits, leading to an outcome-driven style of decision-making at the expense of overlooking lesser measurable factors. The selection preferences of this group suggest that, even though foundational agricultural training is sufficient to identify key functional traits, it might have a shortfall in highlighting nuanced factors that could contribute to the overall performance.

Managers with professional-level education (BSc or MSc) showed comprehensive preferences, in contrast to their college-level mates. SCLH and BCS were strongly preferred. Additionally, this group exhibited a strong preference for STN, SCLS, and LS, suggesting a more holistic approach to decision-making. Higher learning may encourage a broader spectrum of consideration for both performance and physiological parameters when selecting a nurse sow. Enhanced formal training typically correlates with better analytical thinking and multidimensional assessment (Thornhill-Miller et al., 2023). Additionally, the fact that LS was preferred among this group shows that they are conscious of lactation physiology. SB was unfavored, similar to the college group. There seem to be a persistent undervaluation of behavioral attributes in farming systems, despite data linking sow behavior to piglet survival. However, this group represents a well-rounded selection behavior that blends practical productivity with physiological awareness, probably influenced by their level of training.

Finally, in relation to herd size, farm managers managing herds of up to 5000 sows demonstrated a wider and inclusive pattern of preferences favoring all six attributes (Table 6). SCLH, BCS, and SCLS were the most important factors. SB also turned out to be a highly favorable factor in this group, which substantially deviates from other demographic groups, where behavioral traits failed to reach significance. Managers in this group may be more sensitive to behavioral cues. Farm size has a direct impact on operational outcomes such as productivity, efficiency and resource management (Knox et al., 2013). STN and LS's preference suggests that these attributes are not overlooked. The comprehensive selection behavior seen in this group likely strikes a balance between physiological features, performance indicators, and behavioral concerns.

Farm managers managing extra-large herds of >5000 sows preferred SCLH and BCS. The importance of piglet health and sow condition is guaranteed in all herd groups. SCLS remained significant. However, the logistical challenges of overseeing extremely large herds may have contributed to the lack of relevance of the remaining traits. These results are partly in line with those of Baxter et al. (2013), where decisions in large-scale pig operations frequently give precedence to quantifiable, highly significant health and production traits over subtle indicators that could be more difficult to monitor at scale. Above all, in large-scale operations, automation might prioritize measurable metrics above observable parameters.

## 5 Limitation

The most likely limitation of online survey studies is the willingness of the intended participants to participate (Andrade 2020). Furthermore, in the context of our study, this could be coupled with the fact that large-scale farm operations always have busy schedules that could hinder manager’s participation. However, our sample size was consistent with recommendations made by Louviere et al. (2015) and de Bekker et al. (2015) for discrete choice experiments. Another limitation is that individual managers could belong to multiple subgroups simultaneously, which may affect the interpretation of subgroup specific preferences, and hence should be interpreted with caution.

## 6 Conclusion

This study aimed to investigate managers preferences in selecting nurse sows. Managers appeared to place more importance on health-related characteristics over behavioral traits. Farm managers can use these findings to improve nurse sow selection and guarantee more successful piglet fostering. The BWS approach employed in this study may help prioritization of sow attributes by producers. The conditional logit model proved to be a strong analytical method for analyzing complex livestock decision-making processes. This methodology may be extended to other production systems with larger respondents and varied group profiles. These findings have important implications for pig husbandry such that considering managers' preferences in formal decision-making might improve the applicability and adoption of sow management suggestions.

## Ethics statement

The Research Ethics Committee of the University of Debrecen, Hungary approved the research with approval number GTK-KB 011/2025. The study was also part of a wider project investigating the use of nurse sows in swine production.

## Funding

This study was funded by the Tempus Public Foundation under the Stipendium Hungaricum Scholarship SHE-102446-004/2022 for JMO.

## CRediT authorship contribution statement

**Joab Malanda Osotsi:** Writing – review & editing, Writing – original draft, Methodology, Investigation, Formal analysis, Data curation, Conceptualization. **Renata Knop:** Writing – review & editing, Writing – original draft, Visualization, Methodology, Conceptualization. **Samira Chatila:** Writing – review & editing, Writing – original draft, Visualization. **Pedro Urriola:** Writing – review & editing, Writing – original draft, Visualization. **Peter Balogh:** Writing – review & editing, Writing – original draft, Visualization, Validation, Software, Methodology, Investigation, Formal analysis, Data curation, Conceptualization. **Gabriella-Novotni-Danko:** Writing – review & editing, Writing – original draft, Visualization, Validation, Supervision, Resources, Project administration, Funding acquisition, Data curation, Conceptualization.

## Declaration of competing interest

The authors declare that they have no known competing financial interests or personal relationships that could have appeared to influence the work reported in this paper.

## Data Availability

Data are available within the manuscript and its supplementary files.

## Appendix Supplementary materials

Image, application 1
