# Supplementary material for: Investigating attributes for selecting nurse sows in swine herds of Minnesota, USA, using Best-Worst Scaling analysis
Source: Vet Anim Sci. 2026 Apr 12;32:100656. doi: 10.1016/j.vas.2026.100656 (PMC13098455; doi:10.1016/j.vas.2026.100656)
Supplement: Supplementary file 1 [file mmc1.pdf]

Nurse sow selection in swine farms /Selección de cerdas nodrizas en explotaciones porcinas

This survey is aimed at understanding the factors that contribute to nurse sow selection in pig farms.The study is contributing to a larger project investigating the use of nurse sows in swine production.This research is being contacted by Joab Malanda Osotsi, a PhD student at University of Debrecen Hungary.Kindly help filling this short survey in order to achieve the overall aim of the research.
The survey is anonymous.
Thank you

(Esta encuesta tiene como objetivo comprender los factores que contribuyen a la selección de cerdas nodrizas en las explotaciones porcinas.El estudio contribuye a un proyecto más amplio que investiga el uso de cerdas nodrizas en la producción porcina.Esta investigación está siendo contactada por Joab Malanda Osotsi, estudiante de doctorado en la Universidad de Debrecen Hungría.Por favor, ayude a rellenar esta breve encuesta con el fin de lograr el objetivo general de la investigación.
La encuesta es anónima.
Gracias)

1. SOCIO DEMOGRAPHIC QUESTIONS
(CUESTIONES SOCIODEMOGRÁFICAS)
1.What is your age in years?
(1.¿Cuál es su edad en años?)

Mark only one oval.

- ◯ 18-30
- ◯ 31-40
- ◯ 41-50
- ◯ 51-60
- ◯ Above 60 (Más de 60)

2. 2. What is your gender ?
(2. ¿Cuál es su sexo?)

Mark only one oval.

- Female (Mujer)
- Male (Hombre)
- Non binary (No binario)

3. 3.How many years have you been working with pigs?
(3.¿Cuántos años lleva trabajando con cerdos?)

Mark only one oval.

- Less than 1 year (Menos de 1 año)
- Between 1 and 2 years (Entre 1 y 2 años)
- Between 2 and 5 years (Entre 2 y 5 años)
- Between 5 and 10 years (Entre 5 y 10 años)
- More than 10 years (Más de 10 años)

4. 4. What is your highest level of education?
(4. ¿Cuál es su nivel de estudios más alto?)

Mark only one oval.

- High school (Escuela secundaria)
- Some college (technical training) (Algunos estudios universitarios (formación técnica)
- College degree (Bachelors) (Título universitario (Bachelors)
- Professional degree (Masters) (Título profesional (máster)

5. TECHNICAL QUESTIONS
CUESTIONES TÉCNICAS
1.How many sows are in your farm ?
(1.¿Cuántas cerdas hay en su explotación?)

Mark only one oval.

- Less than 1000 (Menos de 1000)
- Between 1001-2000 (Entre 1001 y 2000)
- Between 2001-3000 (Entre 2001 y 3000)
- Between 3001-4000 (Entre 3001 y 4000)
- Between 4001-5000 (Entre 4001 y 5000)
- Above 5001 (Más de 5001)

6. 2.What type of farrowing system does your farm have?
(2.¿Qué tipo de sistema de parición tiene su explotación?)

Mark only one oval.

- Continuous farrowing system (Sistema de partos continuos)
- Batch farrowing system (Sistema de partos por lotes)

7. 3.On average, how many nurse sows does your farm use per day?
(3.De media, ¿cuántas cerdas nodrizas utiliza su explotación al día?)

Mark only one oval.

- Less than 5 (Menos del 5)
- Between 5-10 (Entre 5 y 10)
- Between 10-15 (Entre 10 y 15)
- Above 15 (Más de 15)

8. BWS QUESTIONS (Please don't select more than one response per column)

PREGUNTAS BWS (Por favor, no seleccione más de una respuesta por columna)

How important are the following factors for you when selecting a nurse sow
1.From the following three factors ,please indicate which one is the Most Important and which one is the Least Important (1 of 7)

(¿Qué importancia tienen para usted los siguientes factores a la hora de seleccionar una cerda nodriza
1.De los tres factores siguientes, indique cuál es el más importante y cuál el menos importante (1 de 7))

Mark only one oval per row.

	Most Important (Más importante)	Least Important (Menos importante)
**Sow current litter health status (Estado de salud actual de la camada de cerdas)**	⬭	⬭
**Parity (Paridad)**	⬭	⬭
**Lactation stage (Estado de lactancia)**	⬭	⬭

9. How important are the following factors for you when selecting a nurse sow
2.From the following three factors ,please indicate which one is the Most important and which one is the Least Important (2 of 7)

(¿Qué importancia tienen para usted los siguientes factores a la hora de seleccionar una cerda nodriza
2.De los tres factores siguientes, indique cuál es el más importante y cuál el menos importante (2 de 7))

Mark only one oval per row.

	Most Important (Más importante)	Least Important (Menos importante)
**Sow current litter size (Tamaño actual de la camada de cerdas)**	⬭	⬭
**Parity (Paridad)**	⬭	⬭
**Sow behaviour (Conducta de las cerdas)**	⬭	⬭

10. How important are the following factors for you when selecting a nurse sow
3.From the following three factors ,please indicate which one is the Most important and which one is the Least Important (3 of 7)

(¿Qué importancia tienen para usted los siguientes factores a la hora de seleccionar una cerda nodriza
3.De los tres factores siguientes, indique cuál es el más importante y cuál el menos importante (3 de 7))

Mark only one oval per row.

	Most Important (Más importante)	Least Important (Menos importante)
**Sow current litter size (Tamaño actual de la camada de cerdas)**	◯	◯
**Sow teat number (Número de pezones de la cerda)**	◯	◯
**Lactation stage (Estado de lactancia)**	◯	◯

11. How important are the following factors for you when selecting a nurse sow
4.From the following three factors ,please indicate which one is the Most important and which one is the Least Important (4 of 7)

(¿Qué importancia tienen para usted los siguientes factores a la hora de seleccionar una cerda nodriza
4.De los tres factores siguientes, indique cuál es el más importante y cuál el menos importante (4 de 7))

Mark only one oval per row.

	Most Important (Más importante)	Least Important (Menos importante)
**Sow current litter health status (Estado de salud actual de la camada de cerdas)**	⬭	⬭
**Sow behaviour (Conducta de las cerdas)**	⬭	⬭
**Sow teat number (Número de pezones de la cerda)**	⬭	⬭

12. How important are the following factors for you when selecting a nurse sow 5.From the following three factors ,please indicate which one is the Most important and which one is the Least Important (5 of 7)

(¿Qué importancia tienen para usted los siguientes factores a la hora de seleccionar una cerda nodriza
5.De los tres factores siguientes, indique cuál es el más importante y cuál el menos importante (5 de 7))

Mark only one oval per row.

	Most Important (Más importante)	Least Important (Menos importante)
**Body Condition Score (Puntuación de condición corporal)**	⬭	⬭
**Parity (Paridad)**	⬭	⬭
**Sow teat number (Número de pezones de la cerda)**	⬭	⬭

13. How important are the following factors for you when selecting a nurse sow
6.From the following three factors ,please indicate which one is the Most important and which one is the Least Important (6 of 7)

(¿Qué importancia tienen para usted los siguientes factores a la hora de seleccionar una cerda nodriza
6.De los tres factores siguientes, indique cuál es el más importante y cuál el menos importante (6 de 7))

Mark only one oval per row.

	Most Important (Más importante)	Least Important (Menos importante)
**Body Condition Score (Puntuación de condición corporal)**	⬭	⬭
**Sow behaviour (Conducta de las cerdas)**	⬭	⬭
**Lactation stage (Estado de lactancia)**	⬭	⬭

14. How important are the following factors for you when selecting a nurse sow 7.From the following three factors ,please indicate which one is the Most important and which one is the Least Important (7 of 7)

(¿Qué importancia tienen para usted los siguientes factores a la hora de seleccionar una cerda nodriza
7.De los tres factores siguientes, indique cuál es el más importante y cuál el menos importante (7 de 7))

Mark only one oval per row.

	Most important (Más importante)	Least important (Menos importante)
**Body Condition Score (Puntuación de condición corporal)**	⬭	⬭
**Sow current litter size (Tamaño actual de la camada de cerdas)**	⬭	⬭
**Sow current litter health status (Estado de salud actual de la camada de cerdas)**	⬭	⬭

This content is neither created nor endorsed by Google.

Google Forms
